# γδ T Cells in Antimalarial Immunity: New Insights Into Their Diverse Functions in Protection and Tolerance

**DOI:** 10.3389/fimmu.2018.02445

**Published:** 2018-10-23

**Authors:** Kathleen W. Dantzler, Prasanna Jagannathan

**Affiliations:** Department of Medicine, Stanford University, Stanford, CA, United States

**Keywords:** γδ T cells, Vγ9Vδ2 T cells, *Plasmodium*, cytotoxicity, cytokines, immunological memory

## Abstract

Uniquely expressing diverse innate-like and adaptive-like functions, γδ T cells exist as specialized subsets, but are also able to adapt in response to environmental cues. These cells have long been known to rapidly proliferate following primary malaria infection in humans and mice, but exciting new work is shedding light into their diverse functions in protection and following repeated malaria infection. In this review, we examine the current knowledge of functional specialization of γδ T cells in malaria, and the mechanisms dictating recognition of malaria parasites and resulting proliferation. We discuss γδ T cell plasticity, including changing interactions with other immune cells during recurrent infection and potential for immunological memory in response to repeated stimulation. Building on recent insights from human and murine experimental studies and vaccine trials, we propose areas for future research, as well as applications for therapeutic development.

## Introduction

γδ T cells are unconventional T lymphocytes that are increasingly being appreciated for their unique role in integrating the innate and adaptive arms of the immune system. Comprising approximately 2–5% of peripheral blood T cells in healthy adults, they can uniquely recognize a broad range of antigens without the need for major histocompatibility complex (MHC) and can both establish and regulate the inflammatory response. Many of their individual functions—including production of pro-inflammatory cytokines, cytotoxic killing, antigen presentation, promotion of dendritic cell maturation, B cell help, recruitment of other immune cells, and secretion of growth factors—are shared with other immune cell types. However, a unique combination of antigen specificity, tissue distribution, kinetics and functional properties enable γδ T cells to play an essential role in human immunity (1).

Long known to rapidly increase in number following systemic bacterial or parasitic infection (2–5), γδ T cells may also be important in mediating protection against recrudescence and/or reinfection, particularly in the context of malaria (6–9). Recent investigations of γδ T cell function during this disease caused by parasites in the *Plasmodium* genus are providing new insight into the processes underlying acute responses, as well as protection during chronic or recurrent infection. Despite progress in reducing worldwide incidence of malaria over the last decade, malaria remains a major global health problem, accounting for almost 500,000 deaths annually, predominantly in young children and pregnant women in sub-Saharan Africa (10). Improving our understanding of the inflammatory and immunoregulatory roles of γδ T cells during malaria infection may provide opportunities to manipulate this response therapeutically, potentially via combined targeting of γδ T cells and B or T cell immunity as is currently being pursued for cancer. This review will integrate recent advances in understanding the diverse functions and plasticity of these fascinating cells in malaria. We discuss results from recent human and murine studies, including vaccine trials, and propose open areas for future research and development of novel antimalarial therapeutics targeting γδ T cells.

## The unique functional specialization of γδ T cells

Though γδ T cells can carry out diverse innate- and adaptive-like functions, individual cell subsets have more restricted effector properties depending on expression of T cell receptor (TCR) Vγ and Vδ regions and associated tissue location (1). In humans, the Vγ9Vδ2 subset is the most abundant in adult human peripheral blood; approximately 50–90% of circulating γδ T cells express this combination of chains, previously thought to be due to postnatal expansion. However, Dimova et al. recently demonstrated that Vγ9Vδ2 T cells with pre-programmed effector functions were the predominant γδ T cell subset in fetal blood, suggesting that this subset of γδ T cells may be prepared to respond before birth (11). The other major subset of γδ T cells in humans, Vδ1+ γδ T cells, are enriched in mucosal tissues where they sense host stress and stimulate leukocyte responses (12). In mice, γδ T cells are most common in the skin and mucosal tissue (13) and act as the major initial IL-17 producers in various infectious and autoimmune models. Nearly all murine γδ T cells in the epidermal layer of the skin, also known as dendritic epidermal T cells (DETC), express identical γδ TCRs. In other animals like cattle, sheep, and chickens, γδ T cells express highly diverse TCRs regardless of tissue localization (13). These differences between γδ T cell subsets between species are essential to consider when interpreting conclusions from animal models. Subsets of γδ T cells exhibiting different tissue tropism could have adapted to have differential potential for clonal expansion and therefore diverse roles in immunosurveillance.

Differential γδ T cell subsets recognize different ligands; perhaps the best know interaction occurs between the stress-related phosphoantigens (PAgs) and the Vγ9Vδ2 subset (14). PAgs are intermediates of the eukaryotic mevalonate or the prokaryotic non-mevalonate pathway of isoprenoid synthesis; the former includes eukaryotic PAgs that are overproduced in tumor cells [e.g., isopentenyl pyrophosphate (IPP)] while the latter includes PAgs specifically produced by pathogens, such as (E)-4-hydroxy-3-methyl-but-2-enyl pyrophosphate (HMBPP). Importantly, recognition of these antigens is dependent on cell-cell contact involving the TCR but independent of antigen processing via MHC molecules. The potential for diversity in the γδ TCR repertoire is currently under debate, but there is some evidence from deep sequencing of genomic DNA in a few individuals that though the majority of γδ T cells in peripheral blood carry the same germline TCRγ rearrangement, a substantial percentage (20%) have a more diverse TCRγ repertoire (15). Likely, this sequence diversity represents an evolutionary adaptation to bridge the innate and adaptive immune systems: universal sequences shared across individuals likely perform innate-like functions, while the diverse background repertoire plays a more adaptive role, as has been suggested in a recent study describing TCR repertoires within the Vδ2 compartment (16).

Regarding functional attributes of γδ T cells, γδ T cells can play numerous roles in response to infection, including direct anti-microbial roles, recruitment of innate immune cells (e.g., neutrophils, macrophages) and activation of the adaptive immune compartment (14). For example, γδ T cells rapidly expand in response to mycobacterial phosphoantigen (17), mycobacterium infection (18), and vaccination with the tuberculosis vaccine Bacillus Calmette–Guerin (BCG) (18–20). Vγ9Vδ2 T cells can inhibit mycobacterial growth through soluble granyzme A (17, 21), and correlate with clearance of BCG bacteremia and immunity to fatal tuberculosis in BCG-vaccinated macaques (18). In contrast, human immunodeficiency virus (HIV) is associated with a loss of circulating Vδ2+ cells (22) and expansion of Vδ1+ cells that are able to proliferate and produce IFNγ and IL-17 in response to *Candida albicans* (23). Cytomegalovirus has also been shown to induce Vδ2^neg^ populations of γδ T cells, including Vδ1+ cells, and these Vδ2^neg^ cells have antibody-dependent anti-cytomegalovirus activity through a process that is CD16 dependent (24). Finally, γδ T cells display functional plasticity based on pre-programmed features (i.e., class of innate receptor, nature of inflammatory stimuli, and strength of the TCR signal) or more long-lasting effects induced by TCR signaling and environmental cues (1). For example, Vγ9Vδ2 cells responding to *E. coli* can adapt from a primarily cytokine-producing phenotype to a phenotype promoting phagocytosis (25). Clearly, γδ T cells are capable of adopting a variety of anti-microbial functions, but the precise factors influencing this adaptability in specific infections or at different timepoints during infection require further characterization.

The functional attributes and plasticity of the γδ T cell response to malaria resembles responses to other infections in some aspects, but also reflects the unique life cycle and epidemiology of *Plasmodium*. The epidemiology of malaria in many regions leading to recurrent infection over many years, as well as the diversity of mouse models for malaria, provide opportunities to gain insight into how γδ T cells adapt their function following repeated stimulation.

## Importance of γδ T cells during malaria: insights from natural infection and experimental models in humans and mice

**Figure 1:**
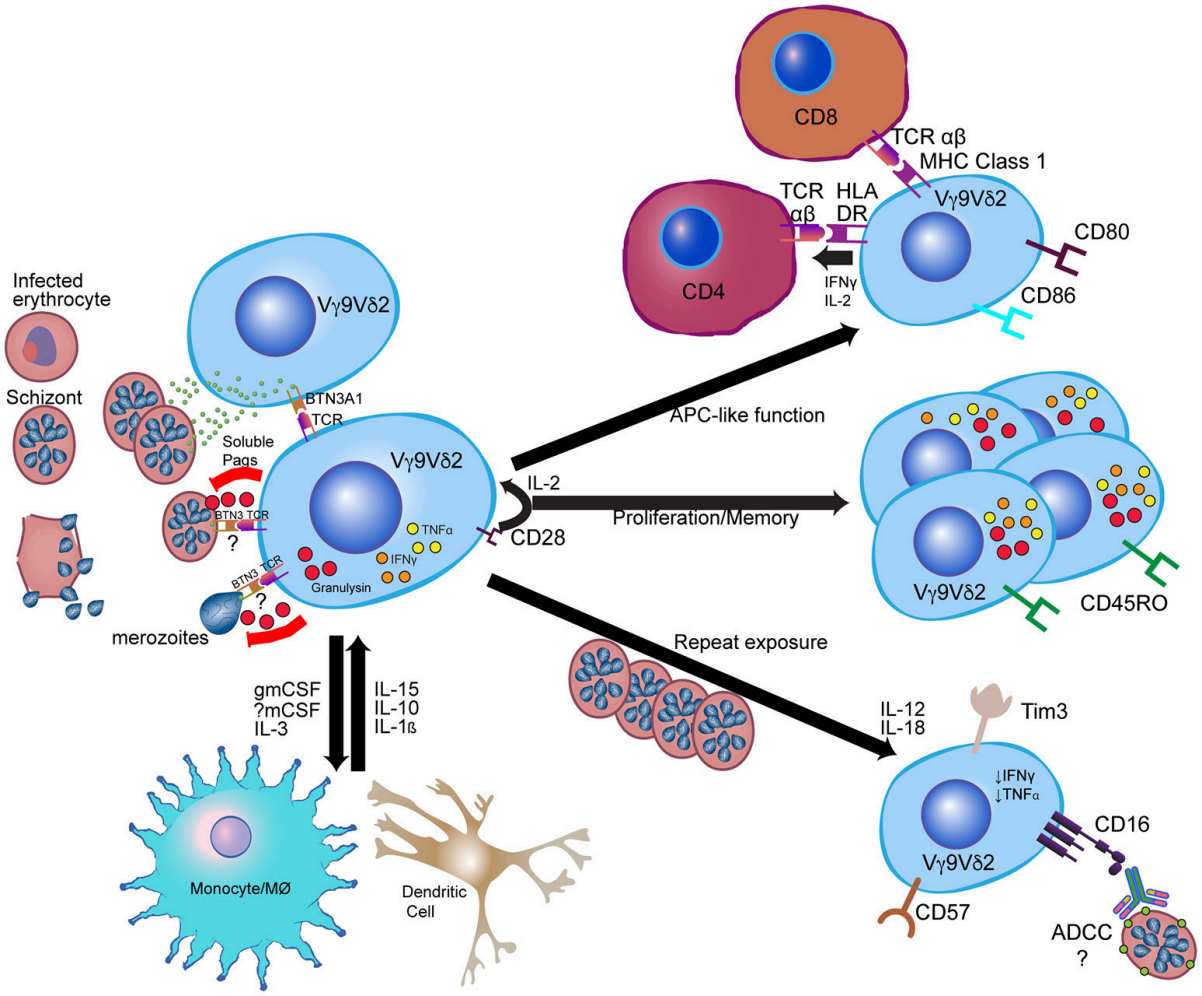
Model for Vγ9Vδ2 γδ T cell response to *Plasmodium falciparum*. Vγ9Vδ2 γδ T cells recognize soluble phosphoantigens released from schizont stage parasites and potentially other iRBC stages. Though precise mechanisms of antigen presentation and recognition remain unclear, phosphoantigens are likely presented to Vγ9Vδ2 TCR via BTN3A1. Other signals, such as CD28 and IL-2 from CD4+ cells and IL-15 from myeloid cells, contribute to Vγ9Vδ2 proliferation and anti-parasitic activity, while Vγ9Vδ2 anti-parasitic activity is dependent on granulysin production. Following activation, Vγ9Vδ2 cells likely influence myeloid cell differentiation and activation through production of myeloid growth factors, and can themselves develop antigen-presenting cell (APC)-like functions such as activation of CD4+ T cells and cross-presentation of antigen to CD8+ T cells. After repeated parasite exposure, cells decrease production of pro-inflammatory cytokines and increase expression of CD16 and immunoregulatory molecules such as Tim-3. CD16 expression may enable alterative functions, such as antibody-dependent cellular cytotoxicity (ADCC). Immune cell images are adapted from the Reactome Icon Library (49).

Whether in children, malaria-naïve adults, or malaria-experienced adults, it has long been known that γδ T cells (in particular the phosphoantigen-responsive Vγ9Vδ2 subset) expand following infection with the most virulent human malaria parasite, *Plasmodium falciparum (Pf)* (Table 1) (26, 29, 30, 47). Furthermore, higher frequencies and malaria-responsive cytokine production of Vγ9Vδ2 cells correlate with protection against subsequent infection in children living in endemic settings (31, 39). These associations, along with cytotoxic, anti-parasitic functions of Vγ9Vδ2 cells observed *in vitro* (48), suggest an important role for these cells in mediating protective immunity to malaria. Though most studies have focused on the Vγ9Vδ2 subset (Figure 1), earlier work suggested that Vδ1+, and not Vδ2+, T cells are expanded in the peripheral blood of individuals from endemic regions (28, 30), similar to what has been described in chronic HIV infection (22). Although this observation may be due to relative loss of circulating Vδ2+ T cells in repeatedly infected individuals rather than expansion of Vδ1+ T cells (34), a recent study demonstrated that non-Vγ9Vδ2, IL-10 producing γδ T cells expand among individuals with uncomplicated malaria (38). Together this suggests that expansion of a non-Vγ9Vδ2 immunoregulatory population of γδ T cells may also contribute to naturally acquired immunity.

**Table 1:** γδ T cell responses to human malaria and associations with clinical outcomes.

**Author, year**	**Country**	**Cohort**	***γδ* T cell subset**	**Impact of malaria exposure on *γδ* T cell activation and function**	**Associations between *γδ* T cell features and clinical outcomes**
**STUDIES OF MALARIA-NAÏVE TRAVELERS**					
Roussilhon et al. (26)	France	Adults; acute *Pf*	All γδ	Expand after infection and remain elevated for months; subset respond *in vitro* to *Pf* schizont extract	
Howard et al. (27)	France	Adults; acute *Pf*	Vγ9Vδ2	*In vivo* exposure and *in vitro* stimulation associated with increased surface expression of APC-associated markers, induce naive αβ T-cell responses, cross present soluble prototypical protein to antigen-specific CD8+ T cells	
**STUDIES OF INDIVIDUALS IN MALARIA-ENDEMIC REGIONS**					
Goodier et al. (28)	Benin	Adults and children	Vγ9+ and Vδ1+	Majority of γδ T cells are Vδ1+; Vγ9+ cells not elevated compared to malaria-naïve controls but do proliferate after *in vitro Pf* stimulation	
Ho et al. (29)	Thailand	Age not reported; acute *Pf*	All γδ	Expand after acute infection and remain elevated for several weeks	
Hviid et al. (30)	Ghana	Children; acute *Pf*	Vδ1+	Increase after treatment and produce pro-inflammatory cytokines	
D'Ombrain et al. (31)	Papua New Guinea	Children	All γδ	Produce IFNγ following *in vitro Pf* stimulation	IFNγ from γδ and αβ T cells associated with immunity to symptomatic infection
Cairo et al. (32)	Cameroon	Neonates	Vδ2+	Placental malaria associated with increased proportions of central memory Vγ2Vδ2 cells in cord blood and altered Vγ2 chain repertoire *ex vivo* or after stimulation	
Stanisic et al. (33)	Papua New Guinea	Children	All γδ	Produce TNF, MIP-1β, and MIP-1α following *in vitro Pf* stimulation	Increased TNF from γδ T cells and monocytes associated with severe malaria
Jagannathan et al. (34)	Uganda	Children	Vδ2+	Repeated infection associated with loss and dysfunction of Vδ2+ cells and increased Vδ2 expression of immunoregulatory genes including Tim3, CD57, CD16	Loss and dysfunction of Vδ2+ cells associated with clinical tolerance to infection
Farrington et al. (35)	Uganda	Children	Vδ2+	Frequencies and function lower and CD16 upregulated among children with high prior malaria exposure; antimalarial chemoprevention associated with enhanced Vδ2+ cytokine production	
Hsu et al. (36)	Malawi	Neonates	Vδ2+	Upregulate PD1 shortly after activation; after engagement of PD1 with PDL1, show dampened TNFα production and degranulation	
Schofield et al. (37)	Papua New Guinea	Children	All γδ	Elevated Tim-3+ γδ T cells across whole cohort; IL-12 and IL-18 contribute to upregulation	Higher proportions of Tim-3+ γδ T cells associated with asymptomatic malaria infection
Taniguchi et al. (38)	Laos	Adults and children; uncomplicated malaria	Non-Vδ2	Expand and produce IL-10 and IFNγ	
Jagannathan et al. (39)	Uganda	Children	Vδ2+	*In vivo* proliferative response attenuated with repeated exposure; repeated infection associated with loss and dysfunction of Vδ2+ cells	Higher pro-inflammatory cytokine production associate with protection from subsequent infection as well as increased odds of symptoms once infected
**VACCINATION STUDIES**					
Teirlinck et al. (40); Roestenberg et al. (41)	The Netherlands	Malaria naïve adults; controlled-human malaria infection (CHMI) + chemoprophylaxis	All γδ	Produce IFNγ, even a year after infection	Long-term functional responses associated with protection against re-infection
Seder et al. (42); Ishizuka et al. (43)	USA	Malaria naïve adults; attenuated PfSPZ vaccination	Vδ2+	Expand after vaccination	Higher frequencies correlate with protection after CHMI
Mordmüller et al. (44)	Germany	Malaria naïve adults; non-irradiated PfSPZ vaccination + chemoprophylaxis	Vγ9Vδ2	Expand in dose-dependent manner and produce IFNγ	
Lyke et al. (45)	USA	Malaria naïve adults; attenuated PfSPZ vaccination	Vδ2+	Cell frequency increase after each vaccination and show activated phenotype	
Zaidi et al. (46)	Mali	Malaria-exposed adults; irradiated PfSPZ vaccination	Vδ2+		Vδ2+ T cells significantly elevated among vaccinees who remain uninfected during transmission season

In malaria-naïve individuals immunized with the attenuated *Pf* sporozoite (PfSPZ) vaccine, expansion and frequency of γδ T cells (again, particularly the Vδ2 subset) was dose-dependent and a better correlate of protection compared to any other cellular immune responses (42, 43, 45). Numbers of memory Vδ2+ T cells similarly correlated with protection in the first PfSPZ trial in a malaria-endemic region; however, additional studies in the mouse led the authors to conclude that these cells were essential for induction of protective CD8+ T cell responses rather than directly exerting effector functions (46). Additional work is needed to further elucidate the mechanism of Vδ2+ γδ T cell-induced protection, as well as to determine whether Vδ2 frequencies could be used as a biomarker for protection in PfSPZ vaccinations in malaria-endemic regions. Furthermore, in a trial immunizing malaria-naïve individuals with non-irradiated PfSPZ combined with chemoprophylaxis (PfSPZ-cVAC), Vδ2+ γδ T cells, including cells expressing memory markers, also expanded in a dose-dependent manner and increased IFNγ expression (44). Together, these data suggest that γδ T cells may be a correlate for both natural and vaccine-induced protection.

**Figure 2:**
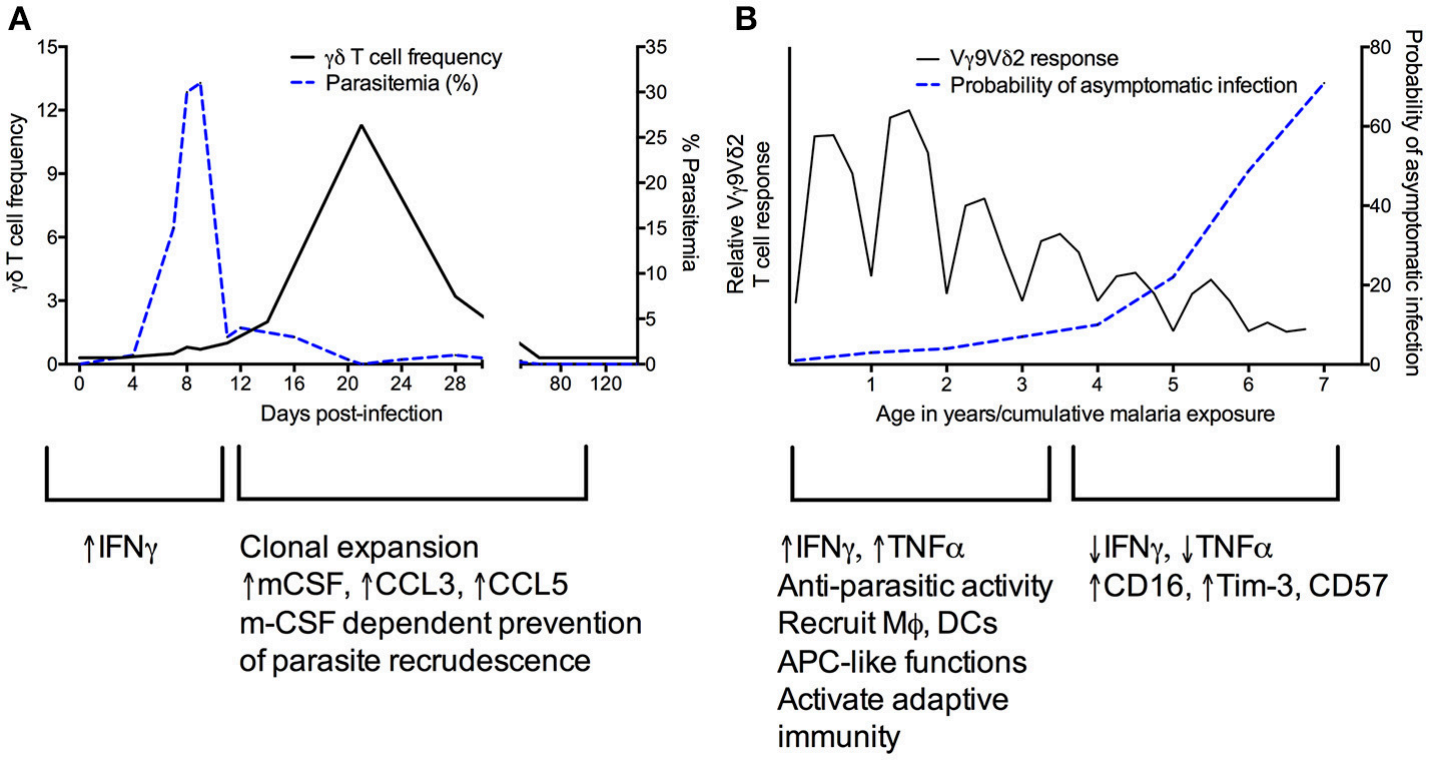
Kinetics of γδ T cell responses during murine *Plasmodium chabaudi* **(A)** and recurrent human *Plasmodium falciparum* **(B)** infections. **(A)** [adapted from Figures 1A,E in Mamedov et al. (9)]. During the acute phase of *P. chabaudi* infection, γδ T cells primarily produce IFNγ. Proliferation of γδ T cells later in infection corresponds with increased production of cytokines such as M-CSF that influence the myeloid compartment, and in parallel, a decrease in parasitemia. **(B)** In human malaria, Vγ9Vδ2 T cells rapidly proliferate and produce pro-inflammatory cytokines during primary *P. falciparum* infection. These cells recruit and activate other immune cells and are able to kill parasites. After repeated infections, Vγ9Vδ2 T cells proliferate less and produce less IFNγ and TNFα while increasing expression of CD16 and regulatory markers such as Tim-3 (34, 35, 39). These changes correlate with an increased probability of asymptomatic infection.

Studies in the mouse model have provided convincing evidence for a role for γδ T cells in directly or indirectly mediating killing of blood-stage and/or liver-stage parasites and preventing parasite recrudescence. However, major differences in γδ clones between mice and humans are an important caveat, as are differences between murine *Plasmodium* strains (Table 2). No subset corresponding to the human Vγ9Vδ2 subset exists in mice and most early studies examined all γδ T cells without regard to antigen specificity. In mice infected with the *Plasmodium chabaudi* parasite, γδ T cells expand by 10-fold (57, 58). Mice deficient in γδ T cells experience higher parasitemia during acute *P. chabaudi* infection, as well as substantial parasitemic recrudescence (6–8). Depletion of γδ T cells during chronic *P. chabaudi* infection in B cell-deficient mice also resulted in significantly worsened parasitemia (56). An exciting recent study demonstrated that γδ T cells expand and become activated in later stages of *P. chabaudi* malaria (Figure 2A)—much later than CD4+ and CD8+ αβ T cell activation—even when acute stages are cleared early by drug treatment. This clonal expansion of γδ T cell occurred primarily in murine blood, spleen, lung and liver, and effectively prevented late-stage parasite recurrence (9).

**Table 2:** Associations between γδ T cells and protection in experimental *Plasmodium* infection models in mice.

**Species**	**Strain**	**Characteristic**	**Finding**	**References**
*Plasmodium yoelii*	Py17X (PyNL)	Non-lethal	• γδ T cells not essential for clearance of blood-stage parasites but do contribute to control of liver stages	Tsuji et al. (50); McKenna et al. (51)
*Plasmodium berghei*	ANKA	Lethal	• Mice with γδ T cells depleted by monoclonal antibody are protected from cerebral malaria • γδ T cells affect CD8α+ dendritic cells in the liver, antigen-specific CD8+ T cell responses in the liver and spleen, and development of protective immunity	Yañez et al. (52); Zaidi et al. (46)
*Plasmodium berghei*	XAT	Non-lethal	• γδ T cells essential for parasite clearance • CD40 signaling between γδ T cells and dendritic cells contributes to control of parasitemia • γδ T cells contribute to humoral immunity	Inoue et al. (53); Inoue et al. (54)
*Plasmodium chabaudi*	AS	Non-lethal in C57BL/6 mice	• γδ T cells expand after infection and produce IFNγ • Mice deficient in γδ T cells have higher parasitemia • γδ T cells expanding later in infection protect against parasite recrudescence	Van der Hyde et al. (55); Langhorne et al. (6); Seixas and Langhorne (7); Weidanz et al. (8); Weidanz et al. (56); Mamedov et al. (9)

In the non-lethal *Plasmodium berghei* XAT model, control of parasitemia seems to be at least partially mediated by CD40 signaling and boosting of dendritic cell activation (53). Following vaccination with lethal *P. berghei* ANKA sporozoites, γδ T cells contribute to pre-erythrocytic immunity by recruiting dendritic cells and CD8+ T cells during vaccination (46), but were not required to prevent infection upon blood-stage challenge. However, different results have been obtained utilizing the nonlethal *Plasmodium yoelii* model. In mice lacking αβ T cells, γδ T cells substantially influenced immunity to *P. yoelii* liver stages, but could not rescue immunity to blood stages (50), suggesting that at least in this parasite strain, γδ T cells act as important effectors and their cytotoxicity may become more effective after interaction with CD4+ T cells. This same group showed that mice lacking γδ T cells had significantly higher *P. yoelii* burden in the liver than similarly challenged immunocompetent mice, suggesting a potential role for γδ T cells in the development of pre-erythrocytic immunity (51). These differences could potentially be explained solely by the different murine parasite strains used, as *P. yoelii* irradiated sporozoite vaccination does not induce sterile immunity while *P. berghei* vaccination does. Alternatively, the discrepant results could be explained by differing sporozoite preparations or the depleting monoclonal antibodies used (59, 60).

Finally, murine models have also suggested a role of γδ T cell in disease pathogenesis. Mice depleted of γδ T cells by monoclonal antibody were protected from cerebral malaria (the most severe form of malaria) due to *Plasmodium berghei*, but mice genetically depleted of γδ T cells did not show this effect (52), implying that effective activation of γδ T cells is extraordinarily time-sensitive. Likely, parasite species/mouse model and IFNγ levels at different infection timepoints strongly influence whether γδ T cells contribute to protection or worsened pathogenesis.

## Recognition of *Plasmodium* by γδ T cells

Numerous *in vitro* and *in vivo* studies have aimed to shed light on the mechanisms of γδ T cell activation in response to malaria infection. *In vitro*, human γδ (Vγ9Vδ2) T cells can proliferate in response to *Pf*-infected red blood cell (iRBC) lysates or schizont extract (34, 61–63), iRBC culture supernatants (62, 64, 65) and/or intact iRBCs (33, 48, 63, 66) (Figure 1). Presumably, these cells are responding to phosphoantigens present in *Pf* asexual blood stages (67), as earlier studies suggested that *Pf* sexual stages do not stimulate Vγ9Vδ2 T cells (68), though it was recently demonstrated that secreted phosphoantigens from iRBCs from all developmental stages (including gametocytes) are capable of stimulating Vγ9Vδ2 cells (69). Activation of Vγ9Vδ2 T cells in response to HMBPP has recently been shown to require butyrophilin 3A1 (BTN3A1, CD277), a type I glycoprotein in the B7 family (70). Though the precise molecular mechanisms underlying BTN3A1 essentiality remain controversial, there is now substantial evidence for a model in which the cytosolic B30.2 domain senses and binds to intracellular pyrophosphates and additional adaptor proteins. A resulting spatial redistribution or conformational change of the extracellular BTN3A1 domain is then recognized via an unknown mechanism by the Vγ9Vδ2 TCR (71–74). Other signaling pathways for human γδ T cell activation involve the TCR interacting with ligands such as F1-ATPase or endothelial protein C receptor, or additional cell surface receptors such as natural killer group 2 member D (NKG2D) receptors or toll-like receptors (TLR). Some murine γδ T cell subsets also appear to have a similar regulation pathway involving non-BTN3A1 butyrophilin-related molecules (75). It is unknown whether such a mechanism occurs during activation of γδ T cells in malaria. In contrast to previous studies suggesting a requirement for cell-cell contact between γδ T cells and parasites in initiating activation (48, 76), Guenot et al. demonstrated that at least for intact iRBCs, BTN3A is not present on the iRBC surface and cell-cell contact is not necessary for Vγ9Vδ2 activation (it may still be required for merozoites) (77). Rather, soluble molecules with characteristics of phosphoantigens seem to be released at the time of *Pf* egress from red blood cells (RBCs), leading the authors to hypothesize that Vγ9Vδ2 activation occurs via presentation by other γδ T cells primarily in microvessels and in the red pulp of the spleen where later stage iRBCs sequester. It will be important to evaluate evidence for this theory, as well as to assess whether myeloid or other cells can present antigen to γδ T cells.

## Proliferation, cytokine, and cytotoxic response of γδ T cells in response to *Plasmodium*

The rapid proliferation of γδ T cells in response to malaria infection likely depends on cytokine signaling and interaction with other immune cells (Figure 1). Proliferating γδ T cells can then inhibit parasite growth *in vitro* (61, 78, 79). Intact iRBCs seem to generally be more effective than lysed iRBCs at inducing γδ T cell expansion; however, lysed parasites can be made more effective with the addition of IL-2 (63). Similarly, activated CD4+ T cells may be required in the absence of IL-2 but are unnecessary when exogenous IL-2, IL-4 or IL-15 is added (64, 80). Proliferation of γδ T cells in mouse spleen during chronic *P. chabaudi* infection appeared to depend on cytokines produced by CD4+ T cells (55). Additional molecules identified as required for IL-2-mediated survival and proliferation include the Ig superfamily receptor CD28 [demonstrated both with human samples and in the *Plasmodium berghei* mouse model (81)] and monocyte-derived cytokines, such as IL-10, IL-12, and IL-1β (82), which also could increase cytokine production by γδ T cells.

During malaria infection, γδ T cells are a major source of IFNγ (Figures 1, 2), which is the cytokine most commonly associated with protection (83). Early IFNγ production has been associated with protection from clinical malaria in some cohorts (31, 84) but associated with worsened symptoms in others (33). There is some debate over whether γδ T cells or NK cells are the predominant source of IFNγ; several authors have suggested that γδ T cells expressing NK cell receptors form the primary source of IFNγ (33, 66, 85) whereas others have argued that γδ T cells are important producers of TNFα but not IFNγ (86). These differential results could be due to differences in timepoints, donors, parasite strains, surface markers used to differentiate cell populations, or immune cell activation conditions; further research, particularly in defining the impact of diversity in γδ T cell numbers and TCR repertoires on heterogeneity of responses, is needed. It is also possible that γδ T cells are required via an unknown mechanism for effective cytokine production by NK cells (86). Which cytokines are produced is likely determined by a combination of factors decided in cell development (e.g., expression of CD27) and epigenetic/transcriptional changes induced by environmental factors (87).

Several *in vitro* studies have provided further insight into the conditions required for γδ T cell effector functions; while IL-2 induces γδ T cell proliferation, for example, IL-15 is needed for anti-parasitic activity (48, 88, 89). Granulysin (but not perforin) released through cytotoxic granules also appears to be required for anti-parasitic activity (48, 76), which is supported by the existence of granulysin-expressing Vγ9Vδ2 cells in patients with malaria (48). Interestingly, Costa et al. showed that while both blood-stage parasites and extracellular merozoites activate Vγ9Vδ2 cells and initiate degranulation, only merozoites trigger anti-parasitic activity by these cells (48). Though the RBC membrane has been suggested to be resistant to granulysin (90), it is currently unclear which parasite stages can be targeted by activated γδ T cells and whether intracellular stages can be targeted via granulysin or other mechanisms.

## γδ T cell roles in stimulating other innate and adaptive immune responses in response to *Plasmodium*

In addition to roles in cytokine production and cytotoxicity, there is increasing evidence that γδ T cells can recruit and stimulate other immune cells, and can adjust pro-inflammatory vs. regulatory effector functions depending on specific host or pathogen factors (for example, cytokines present in the microenvironment) (91, 92) (Figures 1, 2). As described above, non-Vγ9Vδ2 cells were found to proliferate among individuals in a malaria-endemic region and produced the immunoregulatory cytokine IL-10, suggesting expansion of a non-cytotoxic, immunoregulatory population of γδ T cells (38). Furthermore, though γδ T cells have been known to function as antigen-presenting cells (APCs) in other contexts (93–96), this phenomenon was recently demonstrated for the first time in malaria. Vγ9Vδ2 T cells from malaria-infected individuals more highly expressed antigen-presenting and costimulatory molecules, such as HLA-DR, CD80, and CD86, compared with healthy and nonmalarial febrile control subjects (Figure 1) (27). These cells were found to have APC-like functions in response to *in vitro* iRBC stimulation, including activating naive αβ T cell responses and cross-presenting protein to antigen-specific CD8+ T cells (27). Studies in humans have also shown that Vγ9Vδ2 T cells express the myeloid growth factors GM-CSF and IL-3 following *in vitro* stimulation with iRBCs (34). In mice, Mamedov et al. found that a specific γδ T cell clone (TRAVN-1+/V d6.3+) responsible for protection from *P. chabaudi* responded late in infection and prevented parasite recrudescence. Unlike IFNγ-producing γδ T cells responding earlier in infection (9), this clone produced macrophage-colony stimulating factor (M-CSF) and accessory cytokines that influence the myeloid compartment (i.e., CCL5, CCL3) (Figure 2A). Precise differences between IFNγ- and M-CSF-producing γδ T cells, including their direct vs. indirect roles in preventing parasite recrudescence, remain to be defined. Furthermore, whether malaria-induced expression of myeloid growth factors by γδ T cells directly or indirectly influences macrophage function, including epigenetic reprogramming of the myeloid compartment [potentially inducing trained immunity (97)], remains to be determined.

Just as γδ T cells could indirectly influence adaptive immunity through modulation of monocyte function, a parallel process could occur in dendritic cells (DCs). The previously mentioned PfSPZ vaccine trial and corresponding validation in the mouse model suggested that Vδ2+ γδ T cells may be essential for induction of dendritic cell and protective CD8+ responses (46). The absence of γδ T cells during murine vaccination led to dramatically reduced CD8α+ DCs in the liver, impaired antigen-specific CD8+ T cell responses in the liver and spleen, and resulting impaired development of protective immunity (46). These results highlight a possible role for γδ T cells in promoting the migration and/or proliferation of CD8α+ DCs and/or a requirement for cross-talk between γδ T cells and CD8α+ DCs in induction of downstream effector CD8+ T cell responses during PfSPZ vaccination (46). Interestingly, there was no impact of γδ T cell absence on production of antibody targeting the circumsporozoite protein (CSP); however, this does not preclude an effect on antibodies targeting other parasite antigens. Interestingly, γδ T cell expression of CD16, which is known to mediate antibody-dependent cellular cytotoxicity (ADCC) in CMV infection (24) is increased in children in malaria-endemic regions (34, 35), suggesting a potential role for γδ T cells in inciting antibody-mediated parasite killing (Figure 1). Finally, paralleling previous observations during influenza infection (98), recent work in the *P. berghei* mouse model demonstrated a role for γδ T cells in increasing levels of antigen-specific antibodies, Tfh cells, and germinal center B cells via expression of IL-21 and IFNγ early in infection (54). Altogether, evidence suggests that malaria-responsive γδ T cells are able to use diverse direct and indirect (via recruitment of monocytes, dendritic cells, and CD4+ cells) mechanisms to influence effector responses later in infection.

## γδ T cell modulation during recurrent infection

New insights into changing γδ T cell functions during recurrent malaria infection bring up intriguing questions surrounding the relative importance of this modulation in natural immunity to malaria and whether this phenomenon represents a functional or dysfunctional response. Repeated *Pf* infection among Ugandan children was associated with reduced percentages of Vδ2+ γδ T cells, decreased pro-inflammatory cytokine production in response to malaria antigens, and increased expression of CD16 and CD57 and immunoregulatory genes such as HAVCR2 (encoding the inhibitory receptor Tim-3) (34) (Figures 1, 2B). Importantly, though higher Vδ2+ pro-inflammatory cytokine production was associated with protection from subsequent infection, it was also associated with increased odds of having symptoms once infected (39). This suggests that Vδ2+ T cell dysfunction may represent a disease tolerance mechanism allowing for the development of “clinical immunity” to malaria—a decline in symptomatic infections and an increasing proportion of infections that are asymptomatic (34). Alternatively, it is also possible that with repeated exposure, γδ T cell responses gain alternative functional capabilities (i.e., CD16-mediated processes like ADCC).

Regarding mechanisms driving this dysfunction, co-engagement of CD46 and γδ TCR in cells stimulated by HMBPP has been shown to suppress production of IFNγ and TNFα, suggesting that CD46 could be involved in mediating γδ T cell regulation (99). Alternatively, there is evidence for reduced γδ T cell effector function in the setting of Tim-3 expression (37, 39), suggesting that repeated or chronic infection may induce Tim-3 mediated γδ T cell exhaustion, similar to what has been described in Th1 T cells (100, 101) and other innate cells (102). Consistent with this hypothesis, a study found that Tim-3 blockade in murine malaria improved T cell-mediated immunity (103). Schofield et al. recently described that Tim-3+ γδ T cells elevated in children living in malaria-endemic Papua New Guinea were independently associated with asymptomatic malaria infection, consistent with a role for Tim-3 mediated γδ T cell immunoregulation in minimizing symptoms due to malaria (37). Tim-3 expression by γδ T cells in this context was regulated by IL-12 and IL-18 (37). As IL-12/IL-18 can also induce IFNγ production, it will be important to assess the factors and timing differentiating this phenotype from Tim-3 expression, including the role of phosphoantigen.

Additional studies in neonates have highlighted that placental malaria affects the phenotype and repertoire of Vδ2+ lymphocytes in cord blood, potentially lowering the capacity for subsequent Vδ2+ responses to both malaria and other infectious diseases (32). Neonatal Vδ2+ T cells were recently shown to upregulate programmed death 1 (PD1), which when engaged by its ligand, PDL1, decreases TNFα production and degranulation by Vδ2+ cells (36). Intriguingly, PD1 expression by neonatal Vδ2 cells was inversely associated with promoter DNA methylation (36), suggesting a role for epigenetic programming in regulating inflammatory responses. Though much progress has been made in understanding the development of γδ T regulation, further work is needed to more precisely define the underlying mechanisms and to reconcile *in vivo* observations with *in vitro* results showing an increased responsiveness to lysed *Pf* iRBCs after priming with intact iRBCs (85).

## Potential for immunological memory in malaria-responsive γδ T cells

Altered γδ T cell function and upregulated immunoregulatory markers following repeated infection lead to exciting questions concerning the capacity of γδ T cells to develop immunological memory (104), whether similarly to canonical T cell memory or to innate memory. Though γδ T cells have historically been thought of as primarily innate-like, quick-responding cells, there is increasing evidence that these cells are also important at later timepoints during infection and have important adaptive-like functions. In response to CMV, γδ T cells developed a cytotoxic effector/memory phenotype, which in a secondary response led to a faster γδ T cell expansion and a better resolution of infection than the primary response (105). Similarly, distinct primary and recall/memory responses were observed in response to mycobacterial infection in macaques, in which γδ T cell ability to rapidly expand following BCG vaccination correlated with immunity to fatal tuberculosis (18). In controlled human malaria infections in malaria-naive adults, γδ T cells expand late after infection; elevated frequencies of cells expressing effector memory surface markers and enhanced responsiveness to *Pf* stimulation persisted for over 1 year (40). Furthermore, *in vitro* experiments demonstrated that the recall response of γδ T cells post-challenge was independent of other PBMCs (40).

In contrast to adaptive T cell memory, innate-like memory in γδ T cells is an intriguing alternative supported by evidence that monocytes can adapt secondary response to infection based on priming-induced epigenetic reprogramming (106, 107). A recent study has observed this phenomenon, termed “trained immunity”, in monocytes stimulated *in vitro* with *Pf*-iRBCs or from children living in malaria-endemic regions (108). Preliminary evidence showing epigenetic reprogramming at the PD1 locus in neonatal Vγ9Vδ2 cells (36) suggests that a similar process could be responsible for the immunoregulatory phenotype seemingly acquired among γδ T cells after multiple infections (34, 39). Additional questions surrounding memory include the relative importance of adaptive-like vs. innate-like memory cells in protection from malaria, the localization of these cells, and the host and parasite factors influencing their development.

## Directions for future research and application to potential therapeutics

Recent advances in our understanding of the role of γδ T cells in recurrent malaria infection and vaccination highlight numerous open questions and areas for future research (Box 1). Whether γδ T cell activation occurs via cell-cell contact, soluble phosphoantigens released from rupturing schizonts (77) intact iRBCs (69), and/or other parasite stages, and how these cells are precisely able to target and kill *Pf*-iRBC, remains to be elucidated. Though Vγ9Vδ2 cells have been the most studied subset in human malaria, it will be important to determine the role of non-Vγ9Vδ2 cells in infection (38). In addition, evaluating the activation and role of γδ T cells in other important human Plasmodium infections, including *Plasmodium vivax* (109), will benefit eradication programs in diverse settings. Furthermore, a better understanding of human donor variability (66, 77) and parallels and differences between human and murine γδ T cell responses to malaria will enable better translation of observations between the experimental setting and the clinic.

Box 1 **γδ T cells in response to malaria: outstanding questions and areas for future research.**

γδ gd T cell activation and functional specialization.

- What are the precise mechanisms underlying Vγ9Vδ2 T cell activation in response to *Plasmodium falciparum?*
- What is the relative importance of intrinsic vs. extrinsic signals required for γδ T cell activation, proliferation, and anti-parasitic activity?
- How do Vγ9Vδ2 T cells target and kill malaria-infected red blood cells?
- What is the functional role of non-Vδ2+ T cell subsets in human malaria? How are these cells activated?
- What is the role of γδ T cell subsets in the response to non-*Plasmodium falciparum* strains? Given differences in mouse vs. human γδ T cell subsets, how should differences in responses between mouse and human studies be interpreted?

γδ T cell plasticity, memory, and altered function after repeated infection.

- How much diversity exists among malaria-responsive Vδ2+ cells (i.e., do separate “innate-like” and “adaptive-like” subsets coexist?)
- What malaria-induced cellular and microenvironmental cues drive γδ T cell differentiation and plasticity?
- Does dysfunction of Vγ9Vδ2 T cells following repeated malaria represent exhaustion, anergy, or, alternatively, immunologic memory and/or gain of function (e.g., ADCC, antigen presentation)?
- What mechanisms drive Vγ9Vδ2 T cell loss and dysfunction following repeated malaria? (i.e., altered cellular metabolism, epigenetic modifications?)
- Can altered γδ T cell function be reversed or made more functional?
- How do altered γδ T cell functions impact interactions with other immune cells (monocytes, dendritic cells, αβ T cells, B cells, T follicular helper cells)?
- What is the relative importance of innate-like “trained immunity” vs. canonical T cell memory in γδ T cell immunological memory? Does this balance differ in natural infection vs. vaccine-induced exposure?

Applications to novel therapeutics and vaccines.

- Can therapeutic approaches that prevent the development of Vγ9Vδ2 T cell dysfunction enhance parasite clearance?
- Can adjuvant approaches targeting γδ T cells (i.e., BCG, HMBPP, small molecules) influence the development of vaccine-induced malaria-specific humoral or αβ T cell responses?

Current work investigating plasticity of γδ T cells in malaria have exciting implications for our understanding of γδ T cell diversity and memory. For example, if Vγ9+ and Vγ9- Vδ2+ subsets serve distinct “innate-like” vs. “adaptive-like” functions in malaria as has been recently shown in acute CMV (16), this would significantly impact our understanding of differentiation and clonal expansion across age and repeated malaria exposure. Differential cytokine expression in the tissue microenvironment in cancer (91) can induce functional plasticity and differentiation of diverse γδ T cell subsets, but it remains to be determined whether cellular and/or environmental cues similarly drive differential cell fates in malaria. As repeated malaria has been shown to lead to dysfunction of Vγ9Vδ2 T cells, it will be important to assess whether this process represents exhaustion, anergy, or alternatively, immunologic memory and/or gain of function (e.g., ADCC). Mechanisms driving these alterations, including potential epigenetic and/or metabolic perturbations, should be examined (110). In addition, inflammation during recurrent malaria leads to suppression of functional memory B cells (MBC) (111) and expansion of “inferior” T follicular helper (Tfh) cells (112, 113) and “atypical” MBC exhibiting impaired proliferation, cytokine production and antibody secretion (114). Considering the overlapping timing of immunoregulatory phenotypes developing in γδ T cells and adaptive cells, as well as evidence for γδ T cells impacting germinal center reactions in murine malaria (54) and Tfh differentiation during influenza (98), future research should examine the impact of altered γδ T cell function on expansion of MBC and Tfh subsets and interactions with other immune cells.

Novel tools that reduce the immense global burden of malaria by improving natural immunity are urgently needed. Potential therapies involving γδ T cells could directly target γδ T cell responses or could include γδ T cell stimulation in an approach targeting antibody or T cell responses. The former approach could target activating or regulatory molecules or binding affinity/avidity to parasite antigens, induce intracellular accumulation of human Vγ9Vδ2 T cell agonists, and/or stimulate a particular γδ T cell function (e.g., ADCC via increasing CD16 expression). Clinically available compounds performing such functions led to accelerated clearance of *Yersinia pestis* and repair of inflamed tissue in non-human primates (115), and co-administration of Vγ9Vδ2 T cell agonists and IL-2 in cancer patients induced efficient activation of γδ T cells, and ultimately disease stabilization (116, 117). Interestingly, BCG has historically been used as treatment for bladder cancer, potentially by stimulating Vγ9Vδ2 T cells to more efficiently kill cancer cells (118, 119). Future studies investigating the role of BCG or other similar strategies may be useful to enhance responses to malaria and/or other infectious diseases. Therapeutic approaches to prevent γδ T cell dysfunction should also be explored. For example, experimental malaria infection given under chloroquine prophylaxis leads to long-term functional γδ T cell responses associated with protection against re-infection (40, 41), and preventing blood stage infection with highly effective antimalarial chemoprevention was also recently shown to prevent γδ T cell dysfunction in children (35). Drug development could also target inhibitory receptors involved in γδ T cell dysfunction (e.g., Tim-3) or epigenetic pathways involved in modulating plasticity (and possibly trained immunity) of γδ T cells (120).

Regarding approaches using adjuvant γδ T cell stimulation to augment vaccine-induced antibody or T cell responses, one group immunized non-human primates with a subunit vaccine for tuberculosis combined with phosphoantigen. The authors identified a robust γδ T cell response (including development of effector memory surface markers) following primary vaccination but anergy after subsequent boosts (121). In contrast, αβ T cells proliferated after boost vaccinations. These promising results indicate a need for further work aiming to maximize protective responses, whether by preventing γδ T cell anergy or optimizing timing of functional γδ and αβ T cell responses. As the balance between pro-inflammatory and anti-inflammatory responses changes drastically throughout malaria infection, the timing of any of these interventions would be essential to boosting responses without worsening pathology. Such interventions could also be useful for other infections that elicit chronic antigen exposure and/or an exhausted γδ T cell phenotype.

Successful therapies targeting γδ T cells for malaria will likely require a more thorough understanding of (1) functional differences between Vγ9Vδ2 T cell subpopulations, (2) migration and tissue infiltration of Vγ9Vδ2 T cells *in vivo*, (3) cellular interactions in the relevant microenvironment, (4) factors that influence γδ T cell differentiation and exhaustion, (5) γδ T cell detection of and response to metabolic changes in the host, and (6) factors that determine the balance between pro-inflammatory and immunoregulatory responses. The development of tools eliciting long-term, functional γδ T cell responses will be a much-needed addition to the campaign to eliminate and eradicate malaria.

## Author contributions

All authors listed have made a substantial, direct and intellectual contribution to the work, and approved it for publication.

### Conflict of interest statement

The authors declare that the research was conducted in the absence of any commercial or financial relationships that could be construed as a potential conflict of interest.
